# Potential Predictors of Psychological Wellbeing in Elementary School Students

**DOI:** 10.3390/children8090798

**Published:** 2021-09-11

**Authors:** Jun Chen, Xiaozan Wang, Shijun Wu, Jiarong Zhong, Weiyun Chen

**Affiliations:** 1 College of Physical Education and Health, East China Normal University, Shanghai 200241, China; michaelsara2011@163.com (J.C.); xzwang@tyxx.ecnu.edu.cn (X.W.); WSJ151978498@163.com (S.W.); zhongj2@southernct.edu (J.Z.); 2 Research on Physical Education for Adolescents of Shanghai Social Science Innovation Research Base, Shanghai 200241, China; 3 Key Laboratory of Adolescent Health Assessment and Exercise Intervention of Ministry of Education, East China Normal University, Shanghai 200241, China; 4 Research Base of Physical Education and Teaching, Shanghai “LideShuren” Key Research Base of Humanity and Social Science, Shanghai 200241, China; 5 School of Kinesiology, University of Michigan, 830 N University Avenue, Ann Arbor, MI 48109, USA

**Keywords:** soccer skill, fitness, cognitive, wellbeing

## Abstract

Background: This study aimed to investigate the association of elementary school students’ manipulative skill competency, cardiorespiratory fitness, and cognitive function with psychological wellbeing (PWB), as well as whether the association had gender differences. Methods: Participants were 291 fourth-grade students (166 boys vs. 125 girls; mean age = 9.770 years old; SD = 0.584) at two elementary schools from the province of Henan in China. The students’ soccer skills in manipulative skill competency were assessed using the PE Metric Assessment Rubric, cardiorespiratory fitness was assessed by means of the PACER 15 m test, and cognitive function and PWB were assessed using the d2 test of attention and Warwick–Edinburgh Mental Wellbeing Scale, respectively. Data were analyzed with descriptive statistics and multiple linear regression models. Results: The result of linear regression models showed that soccer skills, cardiorespiratory fitness, and cognitive function were collectively associated with PWB for the total sample (*F* (5, 285) = 3.097, *p* < 0.01), boys (*F* (5, 160) = 1.355, *p* < 0.01), and girls (*F* (5, 119) = 2.132, *p* < 0.01). Furthermore, the standardized regression coefficients (*β*) indicated that cardiorespiratory fitness was the only significant contributor to PWB for the total sample (*β* = 0.119, *t* = 2.021, *p* < 0.05), but not for boys and girls. Soccer skills and cognitive function were not individual significant contributors to PWB for the total sample, boys, and girls. Conclusions: Cardiorespiratory fitness was significantly associated with PWB, and there were no gender differences in the relationship of manipulative skill competency, cardiorespiratory fitness, and cognitive function with PWB in elementary school students. This study provides empirical evidence that improving cardiorespiratory fitness is an important intervention strategy to promote elementary school students’ PWB.

## 1. Introduction

It is evidenced that 10–20% of children and adolescents have mental health problems in the world [1]. Specifically, depression and anxiety account for the highest proportion of disability-adjusted life years, reaching 40.5% [2]. According to the World Health Organization, mental health is described “a state of wellbeing in which the individual realizes his or her own abilities, can cope with the normal stresses of life, can work productively and fruitfully, and is able to make a contribution to his or her community” [3] (p. 12). Given that mental disorders in children and adolescents have increased in the past decades [4], it is very important to improve their mental health.

Wellbeing refers to “contentment, satisfaction, or happiness derived from optimal functioning” [5] (p. 70). Furthermore, Ryff [6] indicated that psychological wellbeing (PWB) consists of six dimensions: self-acceptance, positive relations with others, autonomy, environmental mastery, purpose in life, and personal growth. Regular participation in physical activities can help children and adolescents develop their mental health and PWB [7,8]. Studies have shown that the development of children’s motor skill competency, especially manipulative skills, plays an important role in the physical activity participation of adolescents and even adults [9,10,11]. Manipulative skill is defined as the use of hands, feet, or other body parts and objects to control an object (e.g., dribbling, throwing, kicking in sports practice) [12,13,14]. In addition, children’s physical fitness, including cardiorespiratory fitness, muscular strength, muscular endurance, flexibility, and body composition, is related to physical activity [15,16,17].

Previous studies have shown that motor skills were significantly associated with PWB in children and adolescents [18,19,20]. For example, Viholainen et al. [18] found that, among 327 Finland female students aged 12–16, girls’ motor skills, measured using the Development Coordination Disorder Questionnaire, were associated with PWB. Furthermore, Gu et al. [19] found that 141 Hispanic children’s manipulative skills, assessed using the PE Metric Assessment Rubric, were significantly associated with psychosocial health measured by health-related quality of life. In addition, Skinner et al. [21] suggested that students with lower levels of motor skills competency may have negative social and emotional effects, such as being less happy with their lives and placing less value on themselves.

Studies have also found that cardiorespiratory fitness has a significant relationship with PWB. For instance, examining the relationship between physical fitness and mental health (e.g., psychological difficulties including emotional symptoms, conduct problems, hyperactivity, peer relationships, and prosocial behavior) of 1486 Norwegian adolescents, a study found that cardiorespiratory fitness was significantly related to psychological difficulties. However, muscular strength was not related to psychological difficulties when controlling for cardiorespiratory fitness [22]. Another study examined the moderate effects of cardiorespiratory fitness on the relationship between critical life events and health-related quality of life (e.g., PWB, self-esteem, family, friends, and functioning at school) among 378 students aged 6–8. The results showed that students with high levels of cardiorespiratory fitness experienced higher PWB compared to their less fit peers when exposed to elevated stress levels [23].

Cognitive function is defined as “mental processes that contribute to perception, memory, intellect, and action. It provides a core foundation upon which mental health is established.” [8] (p. 2). Moreover, Beddoes et al. [24] indicated that elementary school children’s cognitive functions mainly include five subcomponents, such as attention, working memory, inhibitory control, and cognitive flexibility. Studies showed that lower cognitive function in childhood may be a risk factor for mental health disorders such as generalized anxiety disorder [25,26], psychological distress [27], and depression [26]. In addition, Gale et al. [28] used the data collected during childhood and later life from four Healthy Aging across the Life Course cohorts and examined the relationships among cognitive function in childhood, lifetime cognitive changes, and PWB in older people. The results indicated that 8191 older adults’ cognitive functions in childhood or later life were significantly related to their PWB.

Taken together, motor skills [18,19,20], cardiorespiratory fitness [22,23,29], and cognitive function [26,28,30] are significantly associated with PWB. However, to the best of our knowledge, there has been a lack of studies examining the association of manipulative skill competency, cardiovascular fitness, and cognitive function with PWB in elementary school students. The purpose of this study was twofold: (1) to examine the extent to which manipulative skill competency, cardiovascular fitness, and cognitive function are associated with PWB in elementary school students, and (2) to examine gender differences in the relationships between manipulative skill competency, cardiovascular fitness, and cognitive function and PWB in elementary school students.

## 2. Methods

### 2.1. Participants and Procedures

A total of 291 fourth-grade students (166 boys vs. 125 girls; M ± SD = 9.770 ± 0.584 years old) at two elementary schools from the province of Henan (China) voluntarily participated in this study. Prior to the start of the data collection, we recruited physical education (PE) teachers by using social media to send an overview of the study, including the study purpose, study protocols, and the study outcome assessments, followed by answering their questions and concerns. When the PE teachers agreed to participate, we then sent an invitation letter to the principal of the school, including the study purpose, protocols, and assessment. In the end, two school principals granted permission for their school to participate in this study. Furthermore, the University Institutional Review Board (HUM00149529) approved the study protocols. The students’ parents/guardians signed the informed consent form to allow their children to participate in this study.

Students completed the test regarding manipulative skill competence, cardiovascular fitness, cognitive function, and psychological wellbeing during their regular PE classes. Before data collection, all PE teachers and research assistants received training on testing and evaluation protocols.

### 2.2. Data Collection

#### 2.2.1. Manipulative Skill Assessments

The elementary school students’ manipulative skill competency was assessed using the PE Metrics Assessment Rubrics for soccer dribbling, passing, and receiving skills [31]. The soccer skill assessment rubric has its specific essential dimensions (dribbling, passing, and receiving) and performance indicators (assessment criteria for each level of the rating scale) on 0–4 rating scales [31]. For example, the competence criterion (level 3) for dribbling is “dribbles with control while moving at a slow, consistent jog”, that for passing is: “sends a catchable lead pass to a partner so it can be caught outside the passing lane without a break in the receiver’s stride on at least three passes”, and that for receiving is: “moves forward and outside the passing lane to meet the ball while receiving at least three receivable passes” [31] (p. 120). A total score of nine indicates an overall competent level. The maximum score is 12.

Prior to collecting students’ performance in the soccer dribbling, passing, and receiving skills, the two elementary school PE teachers first received training on use of the soccer skill assessment rubric. Then, during the soccer skills test, the PE teacher demonstrated how to take the test, organized the students into pairs, and gave students two practice trials. Next, the PE teacher used a digital camera to record the soccer skills test. Furthermore, before assessing students’ soccer skills, the two research assistants received 4 h training in assessing and coding students’ performance using the PE Metric Assessment Rubrics. When the inter-rater reliability (IR) [32] reached ≥80% between the two research assistants, they used the PE Metric Assessments Rubric to assess students’ soccer skills. The two research assistants watched the recorded video together, but independently assessed students’ soccer skill performance. In this study, Cronbach’s alpha reliability coefficient of soccer skill assessment was 0.896. The result showed that soccer skill assessment had satisfactory internal consistency reliability [33].

#### 2.2.2. 15 m Version of Progressive Aerobic Cardiovascular Endurance Run (PACER) Test

Students’ cardiorespiratory fitness was assessed using the 15 m version of PACER from Fitnessgram [34]. The PACER test is a validated and reliable cardiorespiratory fitness instrument developed by the Cooper Institute [34]. For the 15 m version of the PACER test, a lap is one 15 m distance and the recorded students’ score is the total number of laps. According to gender- and age-specific standards for the 15 m version of PACER test, ≥21 laps for 10 year old boys and girls is classified in the healthy fitness zone (HFZ) [34].

Before the PACER test, the two elementary school PE teachers received training on how to organize the students to take the test, how to set up the testing areas, how to provide the test instructions, and how to instruct the student using the standardized PACER test recording sheet. During the PACER test, PE teachers first explained the testing directions to students, such as “students should run the 15 m distance and cross the line before the beep sounds; at the sound of the beep, students should turn around and run back to the other end; students should keep running until they fail to reach the line before the beep for the second time” [34] (p. 29). Then, the PE teachers organized students into pairs for two practice trials. Next each pair of students took the test and the number of laps was recorded using the testing recording sheet.

#### 2.2.3. D2 Test of Attention

Students’ cognitive function was assessed using the d2 test of attention [35]. This test was designed to assess selective attention and mental concentration. It consist of 14 lines of 47 characters. The test item includes characters “d” and “p” with 1–4 dashes, arranged either individually or in pairs above and below the characters. During the administration of the test, PE teachers first explained the test instructions to students. For example, students were required to scan the document and cross out “d’s” with two dashes (with two on the top, two on the bottom, or one on the top and one on the bottom), with 20 s to complete each line [35]. Then, PE teachers organized students practice crossing out the “d’s” with two dashes in one line, and they asked whether students understood the test task or had any questions. Next, the PE teachers organized students to take the test strictly following the test protocols.

In our study, the total number of items processed (TN), the percentage of errors (E%), and concentration performance (CP) were used as indictors for evaluating cognitive function. TN, E%, and CP in the d2 test are measures of the processing speed, accuracy, and attention/concentration ability, respectively. Previous research results indicated that the d2 test had a high degree of reliability, ranging from 0.710 to 0.980 [35]. and construct validity [36].

#### 2.2.4. Warwick–Edinburgh Mental Wellbeing Scale (WEMWBS)

Students’ PWB was assessed using the WEMWBS test [37]. This 14-item scale was designed to assess participants’ PWB including positive affect, satisfying interpersonal relationships, and positive functioning [37]. Prior to the test, PE teachers first explained how to fill out the questionnaire for students. For example, the students were asked to read the items and choose the best answer that reflected their experience in the past 2 weeks. Furthermore, students were asked to respond to statements such as “I’ve been feeling optimistic about the future” on a five-point Likert scale (1 = never; 2 = rarely; 3 = some of the time; 4 = often; 5 = all of the time). Then, PE teachers organized students to complete the questionnaire. All items were scored positively and the score of each item was summed to yield the total score of the scale. Previous studies showed that the WEMWBS is a reliable and valid scale for assessing mental wellbeing in youth [37,38]. In this study, Cronbach’s alpha reliability coefficient of WEMWBS was 0.861, indicating a high degree of internal consistency.

### 2.3. Data Analysis

SPSS (Version 27.0; IBM Cooperation, Armonk, NY, USA) was used to conduct all data analysis. The participating students were included in the data analysis if they (1) volunteered to participate in the test, and (2) were healthy with no major disease issues. The participants were excluded from the data analysis if they (1) did not complete the tests, or (2) did not complete the questionnaire. Accordingly, 28 students were excluded from the final data analysis. First, descriptive statistics such as mean scores and standard deviations were established for all outcome variables. In addition, to determine levels and proportions of boys’ and girls’ soccer skill assessments and meeting the HFZ in the PACER test, percentages were computed. Second, in order to examine the relationships of manipulative skill competency, cardiorespiratory fitness, and cognitive function with PWB for the total sample, boys, and girls, multiple linear regression models [33] were generated. Subsequently, standardized regression coefficients [33] were analyzed to assess the relative importance of the soccer skills, PACER test, and cognitive function (TN, E%, CP) in predicting PWB (total score) for the total sample, boys, and girls. A significance level of 0.05 was set for all statistical analyses.

## 3. Results

### 3.1. Descriptive Analysis

Table 1 presents the descriptive statistics of soccer skill assessments and the PACER test for the total sample and by gender. A total soccer skills score of 9/12 indicated an overall competent level. As seen in Table 1, 125 (42.955%) students reached the overall competent level or above in the total sample, with 44.578% of 74 boys and 40.800% of 51 girls. The HFZ standards for the 15 m PACER test were used to classify the participants into two groups: the HFZ (≥21 laps) and needs improvement zone (NIZ) (<21 laps). As seen in Table 1, in the total sample, 88 (30.240%) students reached the HFZ, with 39.759% of 88 boys and 17.600% of 22 girls.

Table 2 presents the descriptive statistics of cognitive function (TN, E%, CP) and PWB for the total sample and by gender. Higher scores in TN and CP and lower scores in E% indicate better performance in terms of attention and concentration. As presented in Table 2, according to the norms designed by Brickenkamp and Zillmer [35], the boys’ percentile rank in TN was 98.900% and the girls’ percentile rank in TN was 95.500%, whereas both boys’ and girls’ percentile ranks in E% were 18.400%, and both boys’ and girls’ percentile ranks in CP were 50%. Furthermore, a higher total score of PWB indicates a high level of mental wellbeing [37]. Based on the initial population study (mean = 50.700) [37], our results showed that the students’ mean PWB score (total sample = 50.290; boys = 50.320; girls = 50.240) was very close to the average level.

### 3.2. Associations of Soccer Skills, Cardiorespiratory Fitness, and Cognitive Function with PWB

Table 3 presents the linear multiple regression model 1 with the soccer skills test, 15 m PACER test, and cognitive function (TN, E%, CP) as independent variables and PWB as a dependent variable for the total sample. As seen in Table 3, regression model 1 indicated that these independent variables were overall significantly associated with PWB (*F* (5, 285) = 3.097, *p* < 0.01). They explained 5.200% of the variance in PWB. The results of standardized regression coefficients (β) indicated that the 15 m PACER test was a significant individual contributor to predicting PWB, while soccer skills and cognitive function (TN, E%, CP) did not display significant β weights at the *p* < 0.05 level.

The results of regression model 2 indicated that the soccer skills test, 15 m PACER test, and cognitive function (TN, E%, CP) were significantly associated with PWB for boys (*F* (5, 160) = 1.355, *p* < 0.01). These independent variables explained 4.100% of the variance in PWB for boys. Furthermore, the results of standardized regression coefficients (β) indicated that the soccer skills test, 15 m PACER, and cognitive function (TN, E%, CP) were not significant individual contributors to predicting the PWB for boys. On the other hand, the results of regression mode 3 indicated that the overall soccer skills test, 15 m PACER test, and cognitive function (TN, E%, CP) were significantly associated with PWB for girls (*F* (5, 119) = 2.132, *p* < 0.01). These independent variables explained 8.200% of the variance in PWB. However, the results of standardized regression coefficients (β) indicated that the soccer skills test, 15 m PACER, and cognitive function (TN, E%, CP) were not significant individual contributors to predicting the PWB for girls.

## 4. Discussion

The first aim of this study was to investigate the relationships of manipulative skill competency, cardiorespiratory fitness, and cognitive function with PWB in elementary school students. Regarding manipulative skill competency, our study found that soccer skills were not significantly related to PWB in elementary school students. Our results are not in line with previous studies that found motor skills to be associated with PWB in Finnish adolescents [18] and Hispanic kindergarten children [19]. However, the inconsistent results might be due to population differences, whereby our study’s participants were Chinese children. In addition, the studies did not always use the same assessment tools to assess motor skills. For example, our study used the PE Metric Assessment Rubrics [31] to assess manipulative soccer skills, whereas [18] used a self-reported adolescent version of the Developmental Coordination Disorder Questionnaire [39] to assess fine motor control and general coordination. On the other hand, [19] used the PE Metric Assessment Rubrics to assess kindergarteners children’s locomotor skills (hopping and sliding) and manipulative skills (dribbling with hand and underhand throwing).

Furthermore, our study found that elementary school students with a healthy level of cardiorespiratory fitness showed higher levels of PWB. These results are supported by previous studies [22,40,41,42]. For example, Andersen et al. [42] found that, among 1129 Norwegian school children aged 10, cardiorespiratory fitness was significantly associated with PWB. Rodriguez-Ayllon et al. [40] examined the association between physical fitness (cardiorespiratory fitness and muscular strength) and psychological wellbeing (negative and positive affect, happiness, optimism, self-esteem) in 110 overweight or obese children. The study found that cardiorespiratory fitness was significantly associated with optimism [40]. Moreover, Eddolls et al. [41] found that 576 British adolescents’ cardiorespiratory fitness had direct and indirect relationships with PWB. Our study further supported the evidence that cardiorespiratory fitness plays an essential role in contributing to the PWB of school-aged children. In addition, our study found that 203 (69.760%) students were in the NIZ group. Our study indicated that the majority of elementary school students in the study need to improve their cardiorespiratory fitness. Given the fact that 10–20% of children and adolescents are affected by mental health issues in the world [1], our study suggests that PE teachers should pay more attention to developing students’ cardiorespiratory fitness through embedding developmentally appropriate fitness activities (e.g., dribbling the soccer ball in general space while walking, jogging, or running and using alternating feet to dribble the soccer ball for 1–2 min) into a variety of PE lessons.

Our study showed that cognitive functions (TN, E%, CP) were not significantly associated with PWB in elementary school students. Inconsistent with our results, other studies found that cognitive functions were associated with PWB [26,30]. For example, Llewellyn et al. [30] investigated the relationship between cognitive functions and PWB in 11,234 adults and found that higher levels of cognitive functions (i.e., cognitive speed, attention, delayed verbal memory) were positively associated with higher levels of PWB. Additionally, Hatch et al. [26] examined the association between childhood cognitive ability and adult mental health in 1875 participants. The results showed that higher cognitive ability in childhood was only associated with fewer symptoms of anxiety and depression in women. However, the reasons for the inconsistent results might be that the dimensions of PWB assessment were different. Assessing PWB in this study focused on dimensions including positive affect, satisfying interpersonal relationships, and positive function, whereas the assessment of PWB in [30] included perceptions of control, autonomy, self-realization, and pleasure. In addition, this study used an adult population, while our study’s participants were elementary school students.

The second aim of this study was to examine the gender differences in the relationships of manipulative skill competency, cardiovascular fitness, and cognitive function with PWB. Our results indicated that manipulative skill competency, cardiorespiratory fitness, and cognitive function collectively played a significant role in contributing to PWB for girls and boys. However, neither boys’ nor girls’ soccer skills, cardiorespiratory fitness, and cognitive function were standalone significant contributors to PWB. Our results are not in line with the previous study by Greenleaf et al. [43], who found that cardiorespiratory fitness was related to PWB as a function of self-esteem and depressed moods in 430 middle school girls, but not boys. Furthermore, Shomaker et al. [29] investigated the relationship between depressive symptoms (negative mood, interpersonal problems, ineffectiveness, anhedonia, and negative self-esteem) and cardiorespiratory fitness in 134 obese adolescents. The study found that only boys’ negative mood was significantly associated with poor cardiorespiratory fitness [29].

It is important to note the limitations of the study. First, our study was a cross-sectional design; thus, it is impossible to determine a causal relationship between manipulative skill competency, cardiorespiratory fitness, and cognitive function and PWB. Second, our study only examined the associations of manipulative skill competency, cardiorespiratory fitness, and cognitive function with PWB. How cognitive functions or manipulative skill competency mediates the relationship between cardiorespiratory fitness and PWB should be investigated in future studies. Moreover, this study only tested the elementary school students’ soccer skills as a manipulative skill competency. Future studies may assess other manipulative skills and locomotor skills to examine their relationship with cardiorespiratory fitness, cognitive function, and PWB. This study provides empirical evidence that cardiorespiratory fitness is significantly associated with PWB. Our study suggests that it is important to develop elementary school students’ cardiorespiratory fitness.

## 5. Conclusions

The unique finding of the study was that cardiorespiratory fitness was a significant contributor to predicting PWB in elementary school students, but manipulative skill competency and cognitive functions were not standalone significantly associated with PWB. Furthermore, there were no gender differences in the relationships of manipulative skill competency, cardiorespiratory fitness, and cognitive function with PWB in elementary school students. This study suggests that developing and improving students’ cardiorespiratory fitness can be an effective approach to promoting PWB in elementary school students.

## Figures and Tables

**Table 1:** Descriptive statistics of manipulative skill assessments and PACER test by gender and total sample.

	Soccer			PACER		
	M	SD	# of OCL (% of OCL)	M	SD	# of HFZ (% of HFZ)
Total	7.235	3.471	125 (42.955%)	18.360	8.752	88 (30.240%)
Boys	7.479	3.587	74 (44.578%)	19.990	10.129	66 (39.759%)
Girls	6.912	3.298	51 (40.800%)	16.200	5.859	22 (17.600%)

Abbreviations: PACER = progressive aerobic cardiovascular endurance run; OCL = overall competent level; HFZ = healthy fitness zone; M = mean; SD = standard deviation.

**Table 2:** Descriptive statistics of cognitive function and PWB by gender and total sample.

	TN		E%		CP		PWB	
	M	SD	M	SD	M	SD	M	SD
Total	384.270	115.349	14.954	17.001	100.270	68.995	50.290	10.580
Boys	385.210	117.804	15.873	17.610	97.040	73.959	50.320	10.467
Girls	383.020	112.465	13.733	16.146	104.570	61.820	50.240	10.769

Abbreviations: TN = total number of items processed; E% = percentage of errors; CP = concentration performance; PWB = psychological wellbeing; M = mean; SD = standard deviation.

**Table 3:** Results of the linear regression model using manipulative skill competency, cardiorespiratory fitness, and cognitive function to predict the PWB.

	*R*	*R* ^2^	F	*p*	Beta	t	*p*
Total Sample							
Model 1	0.227	0.052	3.097	<0.01			
Soccer					0.015	0.251	>0.05
PACER					0.119	2.021	<0.05
TN					0.038	0.423	>0.05
E%					−0.041	−0.255	>0.05
CP					0.144	1.041	>0.05
Boys							
Model 2	0.202	0.041	1.355	<0.01			
Soccer					0.035	0.443	>0.05
PACER					0.108	1.359	>0.05
TN					0.033	0.291	>0.05
E%					−0.052	−0.246	>0.05
CP					0.106	0.565	>0.05
Girls							
Model 3	0.287	0.082	2.132	<0.01			
Soccer					−0.007	−0.082	>0.05
PACER					0.161	1.804	>0.05
TN					0.023	0.151	>0.05
E%					−0.001	−0.005	>0.05
CP					0.210	0.989	>0.05

Abbreviations: PACER = progressive aerobic cardiovascular endurance run; TN = total number of items processed; E% = percentage of errors; CP = concentration performance; PWB = psychological wellbeing.

## Data Availability

The data presented in this study are available on request from the corresponding author.
